# Efficacy and safety of prosthetic arthroplasty of the distal radioulnar joint: a systematic review

**DOI:** 10.1177/17531934261415827

**Published:** 2026-03-01

**Authors:** Dimitris Challoumas, Matthew Arnold, Sumedh Talwalkar, Frederik Verstreken, Carlos Heras-Palou, David Warwick

**Affiliations:** 1 School of Infection and Immunity, College of Medical, Veterinary and Life Sciences, University of Glasgow, UK; 2 Wrightington Upper Limb Unit, UK; 3 West of Scotland Trauma and Orthopaedics, UK; 4 AZ Monica Hospital, Belgium; 5 Pulvertaft Hand Centre, UK; 6 University Hospital Southampton NHS Foundation Trust, Southampton General Hospital, UK

**Keywords:** Aptis, distal radioulnar joint, distal ulna, DRUJ instability, Herbert, prosthetic arthroplasty, Scheker, ulnar head

## Abstract

**Introduction:**

This systematic review evaluates the efficacy and safety of prosthetic arthroplasty of the ulnar head and distal radioulnar joint (DRUJ).

**Methods:**

A comprehensive literature search identified studies reporting functional and safety outcomes of DRUJ prosthetic arthroplasty for 10 or more cases. Primary outcomes included function, pain, and implant survival. Secondary outcomes were satisfaction, grip strength, range of pronation–supination, complications and reoperations.

**Results:**

Forty-three case series were included; of those, 25 reported outcomes of semi-constrained bipolar DRUJ arthroplasty with the APTIS prosthesis (*n* = 706 cases) and 18 of unconstrained ulnar head replacement (DRUJ hemiarthroplasty; *n* = 819 cases). Both the APTIS prosthesis and the participating ulnar head prostheses resulted in functional improvements which were statistically significant and clinically meaningful in most studies in all included functional scales. For pain, mean postoperative improvement ranged from 1.2 to 8.0 (weighted mean 4.3 points) in APTIS and from 1.7 to 5 points (weighted mean 2.5 points) in ulnar head prostheses. Weighted mean long-term survival (>7 years) was 89% with the APTIS and 87% with ulnar head prostheses. Reoperations were required in a weighted mean of 25% of cases in the APTIS group and 23% in the ulnar head arthroplasty group.

**Conclusions:**

Both APTIS DRUJ arthroplasty and ulnar head replacement yield substantial functional improvements and good long-term survivorship. However, complication and incidence of reoperation remain high. These findings highlight the need for improved implant design and material innovation to enhance long-term safety and outcomes.

## Introduction

Pathology of the distal radioulnar joint (DRUJ) can arise from various conditions affecting the ulnar head or the sigmoid notch. When surgical intervention is required, options include soft-tissue interposition, excision arthroplasty (e.g. the Bowers, Darrach or Sauvé–Kapandji procedures) and prosthetic replacement (Moore et al., 2022). Although some surgeons still consider excision arthroplasty to be the surgical treatment of choice, it can result in instability, altered forearm mechanics and symptomatic impingement, particularly in active individuals (Gordon et al., 2003; Rampazzo et al., 2015). Prosthetic arthroplasty options include unconstrained hemiarthroplasties (whereby only the ulnar head is replaced) and semi-constrained total replacements, such as the APTIS (Scheker) prosthesis (APTIS Medical, Louisville, KY, USA), which replaces both components of the DRUJ (Moradi et al., 2021). Outcomes appear more favourable when prostheses are used for primary procedures rather than salvage procedures after failed excision arthroplasty (Sauerbier et al., 2013; Willis et al., 2007; Yen Shipley et al., 2009).

Earlier systematic reviews have reported promising survival rates, but also high complication rates. Calcagni and Giesen (2017) reported overall survival rates of 95 and 98% for the APTIS after 5 years of follow-up, with mean complication rates of 21%. Moulton and Giddins (2017) found 45 and 56 month follow-up survival rates of 93 and 97%, respectively, for ulnar head prostheses and bipolar implants (APTIS), with a mean complication rate of 28% in both groups.

Several additional case series have since been published. Our goal was therefore to update the pooled data on the efficacy and safety of ulnar head and DRUJ prostheses in order to inform clinical decision-making and guide future research.

## Methods

This systematic review was conducted in accordance with the PRISMA guidelines (Online Figure S1) and pre-registered on PROSPERO (CRD420251036382) (Page et al., 2021).

### Literature search

A comprehensive search was performed in PubMed, Medline, and EMBASE (via Ovid) from inception to 25 April 2025, using the following keywords: ‘distal radioulnar joint’, ‘DRUJ’, ‘ulnar head’, ‘replacement’, ‘arthroplasty’, ‘APTIS’ and ‘Scheker’. OpenGrey was also searched for unpublished studies. The reference and citation lists of the included studies were reviewed to identify any further eligible publications. After removing duplicates, 611 studies were identified. Two independent reviewers screened the titles, abstracts, and full texts.

### Study selection

The inclusion criteria comprised observational studies (comparative or non-comparative) involving at least 10 adult patients undergoing ulnar head and/or dDRUJ prosthetic replacement and reporting efficacy and/or safety outcomes. We excluded reviews, case reports, case series (fewer than 10 patients, animal or non-clinical studies, and articles written in languages other than English). Silicone implants were excluded owing to historical safety concerns. Two reviewers independently screened the articles, and any disagreements were resolved through discussion. No minimum follow-up time criteria were set, as it was important to capture early postoperative outcomes, particularly complications. The article screening process is summarised in the PRISMA flow chart (Online Figure S2).

### Participants

Patients older than 18 years of age undergoing ulnar head or DRUJ prosthetic replacement for any indication were included.

### Outcomes

The primary outcomes were function, pain, and implant survival. Functional outcomes were assessed using the Disabilities of the Arm, Shoulder and Hand (DASH) scale, the Patient-Rated Wrist Evaluation (PRWE) scale, and the Mayo Wrist Score (MWS). Pain was evaluated using a 0–10 visual analogue scale (VAS). Scores on a 0–100 scale were converted accordingly. Non-standard scales were excluded.

Secondary outcomes included satisfaction, grip strength (in kilograms or as a percentage of the unaffected side), range of motion (pronation-supination), complications, infections (deep or superficial), reoperation rates and radiographic outcomes. Asymptomatic radiographic changes were not classified as complications. All other adverse events, including those not requiring surgery, were counted as complications. Reoperations and revisions were also counted as complications.

### Data extraction

Two independent reviewers (DC and MA) extracted the following data: study and population characteristics, implant types, indications, follow-up, and outcomes. Any discrepancies were resolved through discussion.

### Data synthesis

Weighted means and ranges were calculated when two or more studies reported the same outcome for the same implant type (ulnar head vs. DRUJ). However, owing to heterogeneity across studies (e.g. patient characteristics, comorbidities, and surgical indications), these means should be interpreted with caution.

For VAS pain scores, rest pain was used if both rest and activity pain were reported. Satisfaction data were pooled from studies indicating satisfaction or willingness to recommend the procedure. Complication and reoperation rates were reported as percentages of affected patients. Implant survival was categorized as short-term (less than 4 years), mid-term (4–7 years), or long-term (more than 7 years). ‘Revision’ was defined as the replacement of any non-modular component.

Subgroup analyses were performed when outcomes for different implant manufacturers were available (e.g. Herbert UHP, KLS Martin, Tuttlingen, Germany vs. Avanta uHead, Avanta Orthopaedics, Inc., San Diego, CA, USA).

For missing data relevant to the analyses, attempts were made to contact the corresponding author of articles published within the last 5 years.

### Quality assessment

All included studies were case series, and their quality was assessed using the Joanna Briggs Institute checklist by two independent reviewers (Munn et al., 2020).

### Statistical methods

Weighted means were calculated by summing the mean for each study, multiplied by its sample size and divided by the total sample size. Medians were assumed to be equal to means. Owing to heterogeneity and limited reporting of variability statistics, no statistical tests were performed, and statistical heterogeneity was not assessed. Clinical significance was defined as differences in outcomes exceeding minimum clinically important differences based on the existing literature, as follows: DASH ⩾10.8; PRWE ⩾11.5; MWS ⩾12.9; and VAS pain ⩾1.6 (Challoumas et al., 2023; Franchignoni et al., 2014; Randall et al., 2022; Walenkamp et al., 2015).

## Results

Forty-three studies (45 articles) were included in the present systematic review (Online Table S1). Twenty-five of these studies reported outcomes of semi-constrained distal radioulnar joint (DRUJ) arthroplasty (*n* = 24 APTIS and *n* = 1 Schuurman prosthesis) in 706 patients (mean age 49.6 years; 60% female; *n* = 698 APTIS from 24 studies; and *n* = 19 Schuurman from one study). Nineteen studies reported outcomes of unconstrained ulnar head replacement in 819 patients (mean age 51.6 years; 56% female; *n* = 567 Herbert UHPs in seven studies; *n* = 85 Avanta UHead in three studies; *n* = 20 First Choice Ulnar Head Prosthesis in two studies; *n* = 22 Ascension MCP Pyrocarbon Prosthesis in one study; *n* = 180 mixed implants in four studies; and *n* = 45 unspecified implants in two studies). The Schuurman prosthesis study was excluded from the data set because the prosthesis has been withdrawn from the market. Therefore, all DRUJ results reflect the outcomes of the APTIS prosthesis (Schuurman, 2013). Two studies only included patients undergoing prosthetic arthroplasty of the ulnar head or DRUJ in patients with wrist fusion, so were excluded (Amundsen et al., 2022; Lans et al., 2019). One study included the outcomes of the APTIS and Herbert UHP, while another included the First Choice partial ulnar head prosthesis and the off-licence MCP pyrocarbon prosthesis (Amundsen et al., 2023; Hebel et al., 2024). In both studies, the results were reported separately for the different implants; therefore, these were analysed as separate datasets in their respective categories.

Among the studies that used the First Choice UHP, one used both partial and total prostheses (*n* = 10). Another included only partial prostheses (*n* = 13) and compared them with the Herbert UHP. A third study included six partial prostheses among other total prostheses; and one compared the partial UHP to the off-licence MCP pyrocarbon prosthesis (Aita et al., 2015; Estermann et al., 2022; Hebel et al., 2024).

Publication years ranged from 2006 to 2024, with a mean follow-up period ranging from 12 months to 11 years and 2 months.

The Scheker group published multiple case series, raising concerns about potential patient overlap. The senior author of a previous systematic review contacted Luis Scheker, who confirmed that the only series to include previously published patients was that of Rampazzo et al. (2015). Therefore, we excluded this series from all analyses (Moulton and Giddins, 2017).

Online Table S2 summarizes the results of the study quality assessment using the Joanna Briggs Institute Case Series Tool. Despite some methodological drawbacks, all studies were deemed to have sufficient methodological quality and reporting for inclusion in the analyses.

Tables 1–3 summarize the pooled results for our chosen outcomes, reported separately for the APTIS and for ulnar head arthroplasty prostheses. Online Tables S3–S5 provide a more detailed description of the results, including ranges, statistical and clinical significance, and subgroup analyses for different ulnar head prostheses.

**Table 1.** Function results of the included studies.

		DASH		PRWE		MWS	
		Mean postoperative score (points)	Mean postoperative improvement from baseline (points)	Mean postoperative score (points)	Mean postoperative improvement from baseline (points)	Mean postoperative score (points)	Mean postoperative improvement from baseline (points)
Distal radioulnar joint arthroplasty (APTIS)	Number of studies (prostheses)	9 studies (n = 207)	2 studies (n = 23)	11 studies (n = 358)	1 study (n = 46)	6 studies (*n* = 113)	4 studies (*n* = 84)
	Weighted mean	25	35	30	26	70	24
Ulnar head arthroplasty (all prostheses)	Number of studies (prostheses)	8 studies (*n* = 234)	4 studies (*n* = 124)	4 studies (*n* = 151)	–	6 studies (*n* = 272)	3 studies (*n* = 167)
	Weighted mean (points)	28	20	40	–	62	21

DASH, Disabilities of the arm, shoulder and hand; MWS, Mayo wrist score; PRWE, patient-rated wrist evaluation.

**Table 2.** Pain, satisfaction and grip strength results of the included studies.

		Pain		Satisfaction	Grip strength		
		Mean postoperative score (points)	Mean postoperative improvement from baseline (points)	Mean postoperative score	Mean postoperative score (kg)	Mean postoperative score (% contralateral hand)	Mean postoperative improvement from baseline (kg or % contralateral hand)
Distal radioulnar joint arthroplasty (APTIS)	Number of studies (prostheses)	17 studies (*n* = 505)	14 studies (*n* = 421)	5 studies (*n* = 124)	15 studies (*n* = 446)	9 studies (*n* = 232)	9 studies (*n* = 192)
	Weighted mean	2.1	4.3	87.1%	20.7	76%	8.0kg
Ulnar head arthroplasty (all prostheses)	Number of studies (prostheses)	7 studies (*n* = 291)	9 studies (*n* = 323)	–	8 studies (*n* = 334)	9 studies (*n* = 275)	6 studies (*n* = 225) for kg 3 studies (*n* = 59) for % contralateral hand
	Weighted mean	1.9	2.5	-	21.0	80%	3.5kg 21%

**Table 3.** Supination and pronation range of movement results of the included studies.

		Supination		Pronation	
		Mean postoperative score (degrees)	Mean postoperative improvement from baseline (degrees)	Mean postoperative score (degrees)	Mean postoperative improvement from baseline (degrees)
Distal radioulnar joint arthroplasty (APTIS)	Number of studies (prostheses)	18 studies (*n* = 512)	12 studies (*n* = 340)	18 studies (*n* = 512)	12 studies (*n* = 340)
	Weighted mean	76	13	70	16
Ulnar head arthroplasty (all prostheses)	Number of studies (prostheses)	12 studies (*n* = 460)	11 studies (*n* = 365)	12 studies (*n* = 460)	11 studies (*n* = 365)
	Weighted mean	71	3	61	4

### Efficacy outcomes

#### Function

Both the APTIS prosthesis and the ulnar head prostheses resulted in good postoperative function (APTIS: weighted mean DASH score 25, PRWE score 30 and MWS score 70; ulnar head: weighted mean DASH score 28, PRWE score 40 and MWS score 62). Statistically and clinically significant improvements in mean scores from baseline were reported in most studies (APTIS: weighted mean DASH score 36, PRWE score 34, and MWS score 24; ulnar head: weighted mean DASH score 20, and MWS score 21).

#### Pain and satisfaction

In patients treated with the APTIS prosthesis, the weighted mean postoperative VAS pain score was 2.1, with a weighted mean improvement of 4.3. The weighted mean preoperative pain score in these studies was 6.1 points. The improvement was statistically and clinically significant in the majority of studies.

For ulnar head arthroplasty, the overall postoperative weighted mean VAS score was 1.9, with a weighted mean postoperative improvement of 2.5. The weighted mean preoperative pain score in these studies was 5.7 points. Pain improvement was clinically and statistically significant in all studies and in most studies, respectively.

Regarding satisfaction, the data were only homogeneous for analyses involving APTIS prostheses. The weighted mean satisfaction score (based on responses to the question of whether they would recommend the procedure) was 87%.

#### Pronation – supination

For the APTIS prosthesis, the weighted mean postoperative supination and pronation were 76 and 70°, respectively. The weighted mean postoperative improvement in supination and pronation was 13 and 16°, respectively. Statistical significance for improvement was achieved in around half of the studies.

For ulnar head arthroplasty, the overall weighted mean postoperative supination and pronation were 71 and 61°, respectively. The weighted mean postoperative improvement was 3° for supination and 4° for pronation. In most studies, the difference did not achieve statistical significance.

##### Grip strength

For the APTIS prosthesis, the weighted mean postoperative grip strength was 20.7 kg, which was 76% of the strength of the unaffected hand. The weighted mean improvement in postoperative grip strength was 8 kg. This improvement was statistically significant in most studies.

For ulnar head arthroplasty, the overall weighted mean postoperative grip strength was 21 kg, or 80% of the strength of the opposite hand. The overall weighted mean improvement in postoperative grip strength was 3.5 kg, or 21% of the contralateral hand. This improvement was statistically significant in most studies.

### Safety outcomes

The survival and complication results are summarized in Table 4, and a more detailed description is provided in Online Table S6.

**Table 4.** Pooled survival results of the included studies.

		Survival			Overall complications	Re-operations	Deep infection	Superficial infection
		Short-term (<4 years)	Mid-term (4-7 years)	Long-term (>7 years)				
Distal radioulnar joint arthroplasty (APTIS)	Number of studies (population)	12 studies (*n* = 417)	7 studies (*n* = 233)	2 studies (*n* = 31)	18 studies (*n* = 600)	18 studies (*n* = 600)	*N* = 7 cases across studies (1.1%)	*N* = 24 cases across studies (3.7%)
	Weighted mean	96.3%	98.0%	89.2%	30.2%	24.9%		
Ulnar head arthroplasty (all prostheses)	Number of studies (population)	6 studies (*n* = 88)	9 studies (*n* = 523)	4 studies (*n* = 208)	14 studies (*n* = 640)	17 studies (*n* = 700)	*N* = 7 cases across studies (1.1%)	*N* = 2 cases across studies (0.3%)
	Weighted mean	89.8%	94.6%	87.3%	22.4%	22.6%		

#### Survival

There was significant variability across studies with regard to the definition of ‘survival’, with some authors even counting revision of whole implants within their survival rates (considering only definitive explantation as ‘non-survival’). Therefore, the true rates may be slightly lower. For the APTIS, the weighted mean survival rates were 96% in the short term (up to 4 years), 98% in the midterm (4–7 years) and 89% in the long term (more than 7 years). When all studies with a mean follow-up of at least 5 years were considered, survival ranged from 80 to 100% (weighted mean survival: 95%, seven studies, *n* = 139).

The overall weighted mean survival rate for ulnar head arthroplasty was 90% for up to 4 years of follow-up, 95% for 4–7 years, and 87% for more than 7 years of follow-up. Excluding the study by Hebel et al. (2024) involving partial head implants (including an off-licence pyrocarbon implant), the weighted mean survival at short-term follow-up was 96% (range 90–100%). When all studies with a mean follow-up of at least 5 years were considered, survival ranged from 83 to 100% (weighted mean survival: 93%, 11 studies, *n* = 681). For Herbert UHP, the survival range in studies with a mean follow-up of at least 5 years was 87–100% (weighted mean 97%, four studies, *n* = 338).

#### Overall complications

The weighted mean of complications in patients who underwent an APTIS prosthesis implantation was 30%. The most common complications were extensor tendon tenosynovitis (particularly of the extensor carpi ulnaris (ECU) tendon), screw prominence, heterotopic ossification and screw loosening.

For all ulnar head arthroplasty implants combined, complications occurred at a weighted mean of 22%. The most common complications were recurrent instability and persistent pain.

#### Reoperations

In the APTIS group, the need for reoperation was observed at a weighted mean of 25%. Common reoperations across all studies included extensor tendon tenosynovectomy or tenolysis (mostly of the ECU tendon), revision, excision of heterotopic ossification, shortening of the radius screw and open reduction internal fixation of a periprosthetic radius fracture.

In the ulnar head arthroplasty group, the weighted mean of reoperations was 23%. The most common reoperations included revision of the entire implant, capsulotomy, revision of the head, conversion to an APTIS prosthesis, removal of hardware owing to irritation of the ECU tendon and extensor tenolysis.

#### Infections

Deep infection was reported in seven cases (1%) and superficial wound infection in 24 cases (4%) with APTIS prostheses. In ulnar head arthroplasty, deep infection occurred in eight cases (1%), while superficial infection occurred in two cases (<1%).

##### Radiographic outcomes

Substantial heterogeneity in the reporting of radiographic outcomes meant that weighted means could not be calculated.

For the APTIS implant, the most common radiographic findings were osteolysis/remodelling at the distal ulna just proximal to the ulnar component (always asymptomatic with no required revision reoperations), radial screw loosening (asymptomatic), heterotopic ossification and aseptic loosening (mostly asymptomatic).

In ulnar head arthroplasty, the most common radiographic finding was osteolysis of the distal ulna just proximal to the collar of the prosthesis, followed by sigmoid notch erosion or sclerosis (both present in up to 100% of cases). Both were asymptomatic and did not require revision. Heterotopic ossification rarely required excision. Aseptic loosening was symptomatic and required revision or explantation in around one-third of cases.

#### Subgroup analyses

Seven studies reported the results of subgroup comparisons for APTIS. Three of these studies found that previous procedures negatively affected outcomes. Stougie et al. (2023) reported no complications in patients without prior procedures. Warlop et al. (2021) found superior functional and motion outcomes in patients with fewer prior procedures, while Lambrecht et al. (2022) demonstrated that patients who underwent wrist arthrodesis or reoperation experienced poorer patient-reported outcomes. Similarly, Scheker (2008) found that the group with prior procedures experienced reduced strength postoperatively compared with those without. DeGeorge et al. (2019), conversely, observed no wound-related complications or tendinopathy in patients without prior surgery. In contrast, Bellevue et al. (2018) found no effect of prior procedures on the incidence of complications, and Rampazzo et al. (2015) reported similar functional outcomes in patients with and without prior procedures. However, patients who did not undergo salvage procedures before receiving the APTIS implant experienced greater pain reduction.

Four studies conducted subgroup comparisons for ulnar head arthroplasty. Axelsson et al. (2015) found that grip strength significantly improved in patients who underwent the procedure for post-traumatic arthritis. Yen Shipley et al. (2009) reported no differences in mean wrist strength between those who underwent arthroplasty as a primary procedure and those who underwent it as a salvage procedure. This was partially in agreement with the results of Mehling et al. (2023), who found no difference in outcomes between those with and without prior procedures. However, DASH score improvements favoured those without a wrist arthrodesis. Finally, Sabo et al. (2014) reported poorer outcomes in patients who underwent ulnar head arthroplasty as a salvage procedure following a failed prior procedure, compared with those who underwent the procedure as a primary treatment.

## Discussion

Our review found that both the APTIS and ulnar head arthroplasty prostheses were associated with meaningful clinical improvements and favourable implant survival. However, both types of implant showed high complication and reoperation rates. Deep infections occurred in around 1% of cases. The only outcome that did not show consistent postoperative improvement was the pronation-supination ROM in patients who received ulnar head arthroplasty. Subgroup analyses favoured the Herbert UHP over the overall ulnar head implant group for all outcome measures and complications.

Our results are comparable with those of two earlier systematic reviews (Calcagni and Giesen, 2017; Moulton and Giddins, 2017), which also showed favourable clinical outcomes, good survival and high complications/reoperation rates associated with APTIS and ulnar head prostheses. Our review included significantly more evidence (43 studies vs. 29 and 18 studies) and also provided weighted means for each outcome measure. Regarding other commonly performed procedures for DRUJ pathology, such as Darrach and Sauvé–Kapandji, the postoperative outcomes reported in the literature are similar to those reported in the present review for prosthetic arthroplasty. However, direct comparisons cannot be made, as a large proportion of the population included in our review had undergone multiple prior procedures, including Darrach and Sauvé–Kapandji, on multiple occasions (Lamont et al., 2022). The mean satisfaction rate with APTIS in our review (87%) is higher than the rate reported for Darrach/Sauvé–Kapandji (around 70%), and the latter figure does not include rheumatoid patients. However, the mean reoperation rate for both APTIS and ulnar head prostheses in our review (both >20%) is much higher than that after Sauvé–Kapandji (7%) and Darrach (3%) procedures.

Online Table S7 summarizes the characteristics of the implants for the APTIS and all the participating ulnar head prostheses. The Herbert UHP is currently the only available ulnar head prosthesis in Europe, although it is temporarily unavailable owing to production issues (as of July 2025). The APTIS prosthesis emains the only semi-constrained total DRUJ implant on the market. A recent kinematic analysis revealed altered forearm biomechanics with the APTIS, including reduced radial translation and an altered rotational axis. These changes could contribute to complications such as mechanical failure or periprosthetic fractures (Oonk et al., 2025).

Despite high survival rates, the complication and reoperation rates remain concerningly high, particularly compared with other common arthroplasties (e.g. hip, knee, and shoulder), which have much better safety profiles (Achakri et al., 2023; Chelli et al., 2022; Piper and Nevasier, 2022). These high rates may be due to the complexity of DRUJ pathology, previous surgeries, implant design, materials, surgical technique or a combination of these factors. These findings underscore the importance of shared decision-making. Our pooled data can assist clinicians when discussing risks and benefits with patients and obtaining informed consent. To improve outcomes, future research should focus on optimizing implant design to restore normal forearm kinematics, refining surgical techniques, and considering newer biomaterials that are less irritating to surrounding soft tissues and easier to revise. Another option is to use patient-specific implants in complex cases. Such efforts could help to reduce complications while maintaining or even improving current survival rates.

This review has several limitations, most of which are related to the studies included. Although our literature search was systematic, the lack of variability statistics in many studies prevented us from making formal comparisons between implant types or across time points. We presented weighted means and reported statistical significance as described by the source studies only. Importantly, we assessed clinical significance using minimum clinically important differences for the DASH, PRWE, MWS and VAS pain questionnaires based on the relevant literature. To minimize bias, we excluded studies with fewer than ten patients and those focusing on highly specific populations (e.g. combined wrist arthrodesis). Nevertheless, the certainty of the evidence is low as all of the included studies were retrospective case series with inherent limitations as well as with heterogeneity in patient characteristics, surgical indications, prior procedures and postoperative rehabilitation. We attempted to address this by conducting subgroup analyses where possible and by assessing study quality. However, the lack of randomized or comparative trials limits the strength of our conclusions.

In summary, although current prosthetic options for the DRUJ, such as the APTIS and Herbert UHP systems, provide functional improvement and favourable survival rates, they are also linked to high complication and reoperation rates. Further innovation and higher-quality comparative studies are needed to improve outcomes and inform future clinical practice. Distal radioulnar joint arthroplasty using a prosthesis seems to have a better outcome when performed as a primary procedure, with poorer outcomes when used to salvage a failed prior resection arthroplasty procedure.

## Supplemental Material

sj-docx-1-jhs-10.1177_17531934261415827 – Supplemental material for Efficacy and safety of prosthetic arthroplasty of the distal radioulnar joint: a systematic review Supplemental material, sj-docx-1-jhs-10.1177_17531934261415827 for Efficacy and safety of prosthetic arthroplasty of the distal radioulnar joint: a systematic review by Dimitris Challoumas, Matthew Arnold, Sumedh Talwalkar, Frederik Verstreken, Carlos Heras-Palou and David Warwick in Journal of Hand Surgery (European Volume)
