# Supplementary material for: Efficacy and safety of prosthetic arthroplasty of the distal radioulnar joint: a systematic review
Source: J Hand Surg Eur Vol. 2026 Mar 1;51(7):847–55. doi: 10.1177/17531934261415827 (PMC13269712; doi:10.1177/17531934261415827)
Supplement: sj-docx-1-jhs-10.1177_17531934261415827 – Supplemental material for Efficacy and safety of prosthetic arthroplasty of the distal radioulnar joint: a systematic review [file sj-docx-1-jhs-10.1177_17531934261415827.docx]

**Online Table S1. Main characteristics of the included studies.**

| Author et al. (year) | PMID | Country | Study type | Implant type (ulnar head vs DRUJ) | Implant name (Company) | Population, n=, mean age (range), %F | Indication/Previous procedures | Mean follow-up (range) | Outcomes |
| --- | --- | --- | --- | --- | --- | --- | --- | --- | --- |
| Laurentin-Perez et al. (2008) | 18332015 | USA | Retrospective case series | DRUJ | Scheker (APTIS medical) | N=31, mean 45.5y (18-70y), 55% F | N=17 previous distal ulna resection  N=5 previous DRUJ fusion  N=9 DRUJ arthritis | Mean 5.9y (4-8y) for n=18 who completed all assessments  Remaining n=13 completed mean 3.5y (1-7y) office visits and 6.3y (5-9y) telephone assessments as unable to travel | Pain (NRS 0-5), function (PRWE, DASH), ROM (pronosupination, fl/ext, deviation), grip strength, WB ability, return to work, complications |
| Brannan et al. 2022 | 34266682 | USA | Retrospective Case Series | DRUJ | Scheker Prosthesis (Aptis) | N = 21, mean age 57 (37-73), 61.9% female | Post-traumatic OA (n=6), degenerative OA (n=5), RA (n=4),, lupus (n=2), seronegative arthropathy (n=2), psoriatic arthritis (n=2),  Previous procedures: failed Darrach (n=1)., distal ulna resection (n=8), soft tissue procedures (n=3), previous wrist fusion (n=3) | 41 months (23-72) | VAS, DASH, PRWE, ROM, grip strength |
| Jawahier et al. (2023) | 37530813 | Netherlands | Retrospective case series | DRUJ | Scheker (APTIS medical) | N=50 (48 patients), mean 56y (IQR 50-65y), 60% F | N=6 degenerative arthritis  N=44 post-traumatic arthritis  (N=20 previous procedures – volar plate distal radius fracture, osteotomy, Sauve-Kapandji, TFCC repair, ulnar head arthroplasty, Bowers procedure) | Median 29m (IQR 12-48m) | Function (PRWE), Complications |
| Levina et al. (2023) | 37790829 | USA | Retrospective and Prospective case series | DRUJ | Scheker (APTIS medical) | N=13 (12 patients), mean 60y, 23% F | All degenerative or posttraumatic DRUJ arthritis  N=8 (62% prior procedures), n-3 (23%) prior trauma | Mean 40m (13-61m) | Pain VAS (0-10), Function (DASH), Subjective grip strength, satisfaction, complications |
| Rampazzo et al. (2015) | 26095055 | USA | Retrospective case series | DRUJ | Scheker (APTIS medical) | N=46 (41 patients), mean 32y (18-39y), 66% F | N=41 for pain  N=5 for instability  (N=37 prior procedures) | Mean 61m | Pain VAS (0-10), Function (DASH, PRWE), lifting strength, grip strength, ROM (fl/ext, pronosupination, deviation), complications |
| Lambrecht et al. (2022) | 34861132 | Belgium | Retrospective case series | DRUJ | Scheker (APTIS medical) | N=21 (loss n=2), mean 48y (24-65y), 52% F | All posttraumatic pain/instability  All had prior procedures (mean 4.9 procedures per patient) | Mean 88m (24-172) | Pain (VAS 0-10), function (PRWE, DASH), ROM (pronosupination, fl/ext), grip strength, satisfaction, complications |
| Bizimungu & Dodds (2013) | 24436836 | USA | Retrospective case series | DRUJ | Scheker (APTIS medical) | N=10, mean 56.2y (SD 16.1y), 50% F | Pain and/or instability due to N=7 posstraumatic, n=1 degenrative, n=1 Madelung, n=1 tumour | Mean 5y (SD 1.1, min 2y) | Pain VAS (0-10), grip strength, ROM |
| Martínez Villén et al. (2023) | 37747488 | Spain | Prospective case series | DRUJ | Scheker (APTIS medical) | N=10, mean 46y (35-65y), %F NR | All failed previous procedures (n=5 Sauve-Kapandji, n=1 radial head excision, n=1 Bowers, n=1 K-wiring, n=1 ex-fix) | Mean 9.7 (3-14.7y) | Pain (VAS 0-10), function (Q- DASH, Mayo Wrist Score), ROM (pronosupination, fl/ext, deviation), grip strength, satisfaction, complications |
| Smith et al. (2024) | 38439648 | USA | Retrospective case series | DRUJ | Scheker (APTIS medical) | N=46, mean 52y (28-79y), 59% F | Failed Darrach n=5, Sauve-Kapandji n=2, Achilles interpsotion n=3, distal ulna arthroplasty n=4, Madelung n=2, periprosthetic fracture n=1, RA/inflammatory arthritis n=6, degenerative arthritis n=10, posttraumatic arthritis n=7, failed matched resection n=2, instability n=5  Mean 2 prior procedures | Mean 60w (8-266w) | Pain VAS (0-10), ROM (fl/ext, pronosupination, complications |
| Reissner et al. (2016)  Fuchs et al. (2020) | 27777820  32126291 | Switzerland | Retrospective case series | DRUJ | Scheker (APTIS medical) | N=10 Reissner  N=9 Fuchs  Mean age 50y (21-64y), 50% F | “Osteoarthritis” n=8  Ulnar stump impingement n=2 | Mean 32m (12-60m) Reissner  Mean 6.3y (5-8.3y) Fuchs | Pain VAS (0-10), Function (Mayo Wrist Score, Krimmer score), grip strength, ROM (pronosupination, fl/ext), lifting capacity, complications |
| Stougie et al. (2023b) | 37762755 | Netherlands | Retrospective comparative cohort | DRUJ | Scheker (APTIS medical) | N=12, mean 50y (26-65y), 33.3% F | N=7 DRUJ OA, n=3 ulnar instability after failed ulnar head prosthesis, n=1 ulnar instability after Sauve-Kapandji procedure  All patients had prior procedures, mean 2.9 procedures | Mean 58m (13-113m) | Function (PRWHE, PROMIS), pain VAS (at rest and max), ROM (fl/ext, pronosupination, deviation), satisfaction, grip strength, complications |
| Stougie et al. (2023c) | 39027021 | Netherlands | Retrospective case series | DRUJ | Scheker (APTIS medical) | N=59 (53 patients), median 56y (IQR 48-63), 62% F | Destroyed DRUJ and ulnar instability n=37, primary DRUJ OA without prior procedures n=11, isolated DRUJ OA after previous surgery n=5, RA n=1, non-union after Sauve-Kapandji n=1, dislocation of ulnar head prosthesis n=1, dislocation of APTIS n=3  (Conversion of ulnar head prosthesis n=12, conversion of failed Sauve Kapandji n=6)  Mean 2.0 prior procedures | Mean 51.3m (SD 21.7 min 2y) | Function (PRWHE, PROMIS), pain VAS (at rest and max), ROM (fl/ext, pronosupination, deviation), satisfaction, grip strength, complications |
| Warlop et al. (2021) | 35971467 | Belgium | Retrospective case series | DRUJ | Scheker (APTIS medical) | N=42 (41 patients), mean 47y (25-74y, 83% F | DRUJ pain n=32 (n=26 previous surgery, n=6 primary)  DRUJ instability n=10 (n=8 previous surgery, n=2 primary)  Posttraumatic 50% cases  Mean 3.2 prior procedures | Mean 46m (24-102m) | Satisfaction, function, ROM (fl/ext, pronosupination, deviation), grip strength, key pinch strength, complications |
| Savvidou et al. (2013) | 24436788 | USA | Retrospective case series | DRUJ | Scheker (APTIS medical) | N=36 (35 patients), mean 44y (23-74y), 60% F | “rheumatoid, congenital, degenerative or posttraumatic arthritis of the DRUJ”  92% patients prior surgery | Mean 5y | Pain VAS (0-10) at rest and with activity, Function (DASH, PRWE), lifting strength, ROM (pronosupination), satisfaction, grip strength, complications |
| Kachooei et al. (2014) | 25386579 | USA | Retrospective case series | DRUJ | Scheker (APTIS medical) | N=14 (13 patients), mean 44y (SD 5.8y), 71% F | Instability n=8, posttraumatic arthritis n=2, stiffness n=1, deformity n-1, Ehlers-Danlos n=1 | Mean 12m (2-25m)  Median 60m (2-102m) for PROMs | Pain VAS (0-10), function (DASH, PRWE), satisfaction, ROM (fl/ext, pronosupination), grip strength |
| Schuurman (2013)  Schuurman & Teunis (2010) | 24436843  20843612 | Netherlands | Retrospective case series | DRUJ | Schuurman DRUJ prosthesis | N=19, mean 45y (22-62y), 89% F | N=10 failed Darrach, n=7 failed Sauve-Kapandji, n=1 trauma, n=1 destruction by synovitis | Mean 49m (1-7y) | Pain VAS (0-10), Function (DASH), ROM (fl/ext, pronosupination, deviation), strength (grip, tip, lateral), complications |
| Bellevue et al. (2018) | 29275901 | USA | Retrospective case series | DRUJ | Scheker (APTIS medical) | N=52 (48 patients), mean 47y (18-66y), 48% F | N=29 posttraumatic, n=10 Madelung, n=2 RA, n=11 degenerative OA  Mean 2.6 prior procedures | Median 1.4y |  |
| Jawahier et al. (2023) | 37530813 | Netherlands | Retrospective case series | DRUJ | Scheker (APTIS medical) | N = 50 (48 patients), median age 56, 60% female | Degenerative arthrosis (6), post traumatic (44). Previous procedures: distal radius ORIF (8), correction osteotomy (4), TFCC repair (3), failed ulna head resection (2), failed Sauvè–Kapandji (1), failed ulnar head arthroplasty (1), failed bowers (1) | 29 months | Patient-Related Wrist Evaluation |
| Scheker et al. 2008 | 18984351 | United States | Retrospective Case Series | DRUJ | Scheker (APTIS medical) | N = 49 | NR | 2 years | Grip strength, pain, ROM |
| Scheker and Martineau 2013 | 18984351  **23168033** | United States | Retrospective Case Series | DRUJ | Scheker (APTIS medical) | 1^st^ generation: N = 31  2^nd^ generation: n=35 | NR | 1^st^ generation: 5.9 years (4-8)  2^nd^ generation: 5 years | DASH, PRWE, ROM, Grip strength |
| Amundsen et al. (2022a) | 34446335 | Norway and USA | Retrospective case series | DRUJ | Scheker (APTIS medical)  Conversion of Avanta uHead (Small bone innovations) n = 10 and Ascension first choice n = 1 | N = 11 (10 patients), medan age 47 (30-75), 70% female | All patients had previous porous coated stem ulna head implant. Indications – pain/instability (n-6), pain/impingement (n=2), pain and sigmoid notch wear (n=2), pain and loosening (n=1) | 64 months (15-123) | Pain (not VAS), mayo wrist scores, ROM, grip strength |
| DeGeorge et al. 2017 | 30344019 | USA | Retrospective case series | DRUJ | Scheker (APTIS medical) | N=49 (50 patients), mean age 47.8 +/-1.9, 66% female | Indications:  Chronic pain and instability (n=38), post traumatic arthritis (n=28), DRUJ deformity (n=10)  Previous operations: Ulna head resection (52%), TFCC repair (34%), ulna head replacement (28%), wrist fusion (18%), wrist arthroscopy (18.4%). | 35.8 +/-3.7 months | VAS, ROM, grip strength, |
| Galvis et al. (2014) | 24996676 | USA | Retrospective case series | DRUJ | Scheker (APTIS medical) | N=19 (17 patients), mean 57y (38-85y), 70.6% F | All RA arthritis | Mean 39m (12-79m) | Pain VAS (0-10), ROM (pronosupination), function (DASH, PRWE), satisfaction, lifting capacity, complications |
| Amundsen et al. 2023 | 35550310 | Norway and USA | Retrospective Case Series | Ulna Head and DRUJ | Scheker (APTIS medical) and Avanta uHead UHPs | Aptis n = 53, mean age 51 (21-74), 62.3% female  UHPs n = 102, mean age 50 (19-86), 61.8% female | Aptis: Darrach or Sauve Kapandji (37.7%), ulnar shortening (17%), previous ORIF (20.8%), TFCC debridement (22.6%), DRUJ stabilisation (20.8%), previous wrist fusion (17%)  UHP: Darrach or Sauve-kapandji (25.5%), ulnar shortening (6.9%), previous ORIF (22.5%), hemiresection ulna head (5.9%), TFCC debridement (19.6%), DRUJ stabilisation (20.6%), wrist fusion (19.6%), total wrist arthroplasty (8.8%) | Aptis: 30 months,  UHP: 102 months | Pain, mayo wrist scores, ROM, grip strength |
| Aita et al. (2015) | 27218078 | Brazil | Retrospective case series | Ulnar head | Ascension (First Choice=partial or Modular Ulnar Head=total) | N=10, mean 50.3y (26-64y), %F NR | Post Darrach (n=2), post Sauve-Kapandji n=3, ligament injury n=2, complex fracture n=3 | Mean 16.8m (12-36m) | Function (DASH), Pain VAS (0-10), ROM (pronosupination, complications |
| Van Schoonhoven et al. (2012)  Van Schoonhoven et al. (2000) | 22652179  10811747 | Germany | Retrospective case series | Ulnar head | Herbert UHP (KLS Martin) | N=23, mean 45y (22-65y), 52.2% F | All previous distal ulna resection (n=2 silastic, n=11 Bowers, n=10 Darrach) | Mean 27m (10-43m) and 11y2m (97-158m) – N=16 available for long-term F/U | DRUJ stability, ROM (pronosupination), pain VAS (1-4), satisfaction (1-10), grip strength, complications |
| Willis et al. (2007) | 17275592 | USA | Prospective case series | Ulnar head | uHead (Small Bone Innovations) | N=19 (17 patients), mean 51y (32-75y), 53% F  Uncemented n=13, cemented n=6 | N=5 degenrative OA, N=4 RA/inflame arthritis, N=10 posttraumatic arthritis  Prior surgery N=13, average 2.8 procedures | Mean 32m (26-60m) | Pain VAS (1-4), Function (Mayo Wrist Score), ROM (pronosupination), grip strength, functional satisfaction (1-4), complications |
| Mehling et al. (2023) | 36515709 | Germany | Retrospective case series | Ulnar head | Herbert UHP (KLS Martin) | N=62, mean 49y (18-84y), 45.2% F | Pain and instability due to failed Sauve-Kapandji n=10, Bowers n=37, Darrach n=5, posttraumatic arthritis n=8, degenerative arthritis n=2 (mean 3 prior procedures) | Mean 84.5m (8-206m) | Pain VAS (0-10), Function (DASH, Mayo Wrist score), grip strength, ROM (pronosupination), complications |
| Gvozdenovic et al. (2020) | 33042644 | Denmark | Prospective case series | Ulnar head | uHead (Small Bone Innovations) | N=20, mean 59y (36-80y), 70% F | “Severely painful destruction of DRUJ” – in 3 cases the stability component of uHead was also used | Mean 5y (2-15y) | Pain VAS (0-10), ROM (pronosupination), function (qDASH), satisfaction, grip strength, complications |
| Fernandez et al. (2006) | 16601411 | Switzerland | Retrospective case series | Ulnar head | Herbert UHP (KLS Martin) | N=10 | All failed Sauve-Kapandji | Mean 2.6y (8-74m) | Pain (Fernandez pain score, 0-3), instability (scale 0-3), complications |
| Shipley et al. (2009) | 19730047 | USA | Retrospective case series | Ulnar head | Herbert-Martin (Martin Medizin-Technik, Tuttingen,  Germany) or Avanta (Small Bone Innovations, New York,  NY) | N = 22 (20 patients), mean 47.9, 55% female | 11 patients had history of trauma. 14 patients had prior surgery (Feldon wafer  excision, Bowers hemiarthroplasty interposition, Darrach  procedure, Sauve-Kapandji procedure, arthroscopy, debridement/  repair of ligaments.) | Mean 54.3 months | Verbal analog pain score, modified mayo wrist score |
| Kakar et al. (2012) | 22721459 | USA | Retrospective case series | Ulnar head | Avanta (Small Bone Innovations, Morrisville, PA) | N = 46, mean 48 (19-84), 62% female | Post-traumatic OA (47%), primary OA (15%), DRUJ dislocations (11%), other causes (13%). Previous procedures: Darrach (n=13), wrist ORIF (11), TFCC debridement (n=6), DRUJ stabilisation (n=7), partial wrist arthrodesis (n=5), total wrist arthroplasty (n=3), total wrist arthrodesis (n=2), hemi-resection and interposition arthroplasty (n=2) | 56 months (16-126 months) | Pain, Mayo scores, grip strength, radiological outcomes, |
| Adams et al. (2017) | 28399786 | USA | Retrospective case series | Ulnar head | NR | N = 28, mean 54 (23 – 82), gender not recorded | Primary DRU joint arthritis (14); arthritis secondary to fracture or malunion (10); rheumatoid arthritis (2); and acute ulnar head fracture (2). 12 patients had undergone previous operations on the distal ulna: distal radius fracture fixation (4); Darrach procedure (2); partial ulnar head resectional arthroplasty (3) silicone ulnar head implant (1); and wrist arthrodesis (1). | 4.6 years | Forearm range of motion, patient-rated wrist evaluation scores, pain (non-specific) |
| Axelsson et al. 2015 | 26216078 | Sweden | Retrospective case series | Ulnar head | Herbert UHP (Martin Medizin Technik, Tuttlingen, Germany) | N = 21 (20 patients), mean age 55 (31-74), 50% female | Painful  instability after previous resection arthroplasty (10  wrists), pain due to osteoarthritis (9 wrists), and rheumatoid  arthritis (3 wrists).  Previous surgery: fixation  of distal forearm fractures (2), corrective osteotomy  of the distal radius (2), plate removal (2), ulnar  shortening (2), ulnar styloidectomy (1), triangular  fibrocartilage reinsertions (3), Darrach procedures (10),  stabilizations of unstable ulnar stump (3), total wrist  arthroplasty (3), total wrist arthrodesis (1), arthroscopy  with shaving (1), tendon transfer (1), synovectomy (1),  and neuroma excision (2). | 7.5 years (2-12.5 years) | Pain VAS, Mayo wrist score, DASH, PRWE, ROM |
| Sabo et al. (2014) | 25443169 | UK | Retrospective case series | Ulnar head | 53 patients had Herbert-type prostheses (KLS Martin, Tuttlingen,  Germany), 6 had a First Choice implant (Ascension  Orthopaedics Inc, Austin, TX), 21 patients,  No implant info available. | N = 79 (74 patients), mean age 50 (24-76), 68.9% female | Post traumatic (32), inflammatory arthritis 19), osteoarthritis (12), 3 patients uncertain.  Previous procedures: Arthroscopy 19, ulnar shortening 13, bowers 7, distal radius osteotomy 4, DRUJ stabilisation 2, Darrach procedure 22, Sauve Kapanji 17, removal hardware 11, ECU stabilisation 6, wrist fusion 19, wrist arthroplasty 4 | 7 years | ROM, patient evaluation measure, PRWE, Wrightington wrist score, EQ5D, |
| Warwick et al. 2013 | [24025293](https://pubmed.ncbi.nlm.nih.gov/24025293/) | UK | Retrospective case series | Ulnar head | Herbert prosthesis (KLS Martin, Tuttlingen, Germany) for 52 cases, the uHead™ prosthesis (Avanta Orthopaedics, San Diego, CA, US) for 3 cases and the spherical ulnar head prosthesis (KLS Martin) for 1 case. | N = 56 (52 patients) | RA n = 21, OA n = 18, Failed sauve-kapandki n = 4, failed darrach’s n = 3, ulnar neck fracture n = 2, radius malunion n = 2, ulnar malunion n = 2, Vaughan-jackson syndrome n = 1,benign tumour n = 1, psoriatic arthritis n = 1 | Mean 60 months (1 year to 11 years) | Pain VAS, DASH, |
| Estermann et al. 2022 | 34622695 | Switzerland | Retrospective case series | Ulnar head | First Choice® (Ascension Orthopaedics Inc, Austin, TX, USA) (partial) or Herbert Ulnar Head® Prosthesis (KLS Martin, Tuttlingen, Germany) (total) | N = 23 (22), partial UHI mean age 56 (29-70), total UHI mean 60 (45-75) | Post-traumatic arthritis (13), primary OA (4), distal ulna stump instability (3), RA (2), post traumatic DRUJ instability (1).  Previous surgeries: | Mean 7 years (1.3 to 17) | PRWE, DASH, grip strength, ROM, complications, USS examination |
| Maling et al. 2024 | 38534146 | United Kingdom | Retrospective cohort study | Ulnar head | Herbert ulnar head prosthesis (UHP; KLS Martin, Freiburg, Germany) | N = 232 (219 patients), mean age 64 (25-89), | Primary OA – 107 (47%)  Rheumatoid Arthritis – 55 (24%)  Failed Darrach – 16 (7%)  Post-traumatic OA – 15 (7%)  Vaughan Jackson – 13 (6%)  Failed Sauve Kapandji 7 (3%)  Psoriatic arthritis – 6 (3%)  Instability – 3 (1%)  Trauma – 2(1%)  Crystal arthropathy – 1 (<1%)  Cystic lesion ulnar head – 1 (<1%)  SLE – 1 (<1%) | Mean 5.5 years (1 month – 21 years) | Revisions, complications |
| Herzberg et al. 2010 | 20951906 | France | Retrospective case series | Ulnar head |  | N = 17, 76.5% female | Failed Darrach (4), Sauve-Kapanji (2), RA (5), distal radius malunions (4), giant cell tumour (1), primary OA (1) | Mean 36 months (25 – 63 months) | Pain, ROM, grip strength, sigmoid notch erosion, radiographic loosening |
| Saurbier 2013 | 24436786 | Germany | Retrospective case series | Ulnar head | uHead (n=20)  Herbert (UHP; KLS Martin, Freiburg, Germany) (n=5) | N = 25, mean age 48 (28-75), 24% female | Post traumatic OA, previous ulnar head resection, primary OA | Mean 30 months (2-109 months) | Pain, ROM, grip strength, DASH, Kirmmer, Cooney wrist scores, |
| Fok et al. 2019 | 29934080 | Switzerland and Germany | Retrospective Case Series | Ulnar Head | UHP (spherical UHP; KLS Martin Group, Tuttlingen, Germany) | N = 17, mean age 47 (29-70), 58.8% female | All failed Sauve Kapandji procedures: post traumatic OA (n-15), primary OA (n-1) and dysplastic DRUJ (n-1) | Mean 6 years (4-17) | Pain (Fernandez score), ROM, grip strength, DASH |
| Hebel et al. 2024 | 40151782 | USA | Retrospective Case Series | Ulnar head | First Choice® (Ascension Orthopaedics Inc, Austin, TX, USA) (partial) or Ascension MCP pyrocarbon implant (Ascension Orthopaedics Inc, Austin, TX, USA) | N=32 (N=10 metallic, N=22 pyrocarbon), median age 57y metallic head, 50y pyrocarbon, 70%F | Posttraumatic arthritis, OA, RA, instability, impaction, malunion, meniscal allograft | Mean 37.8 months for metallic head  Mean 25 months for pyrocarbon | Pain, ROM (flexion, extension, pronosupination), grip strength, complications |

**Online Table S2.** Study quality assessment of the included case series using the Joanna Briggs Institute Case Series Assessment tool

|  | Were there clear criteria for inclusion in the case series? | Was the condition measured in a standard, reliable way for all participants included in the case series? | Were valid methods used for identification of the condition for all participants included in the case series? | Did the case series have consecutive inclusion of participants? | Did the case series have complete inclusion of participants? | Was there clear reporting of the demographics of the participants in the study? | Was there clear reporting of clinical information of the participants? | Were the outcomes or follow up results of cases clearly reported? | Was there clear reporting of the presenting site(s)/clinic(s) demographic information? | Was statistical analysis appropriate? |
| --- | --- | --- | --- | --- | --- | --- | --- | --- | --- | --- |
| Adams et al. (2017) | Y | Y | Y | Y | Y | Y | Y | N | Y | Y |
| Aita et al. (2015) | Y | Y | Y | Y | Y | Y | Y | Y | Y | Y |
| Amundsen et al. 2022 | Y | Y | Y | Y | Y | Y | Y | Y | Y | Y |
| Amundsen et al. 2023 | Y | Y | Y | Y | Y | Y | Y | N | Y | Y |
| Axelsson et al. 2015 | Y | Y | Y | Y | Y | Y | Y | Y | Y | Y |
| Bellevue et al. (2018) | Y | Y | Y | Y | Y | Y | Y | N | Y | Y |
| Bizimungu & Dodds (2013) | Y | Y | Y | Y | Y | Y | Y | N | Y | Y |
| Brannan et al. (2022) | Y | Y | Y | Y | Y | N | Y | Y | Y | Y |
| DeGeorge et al. 2017 | Y | Y | Y | Y | Y | Y | Y | Y | Y | Y |
| Estermann et al. 2022 | Y | Y | Y | Y | Y | Y | Y | Y | Y | Y |
| Fernandez et al. (2006) | Y | Y | Y | Y | Y | N | Y | Y | Y | Y |
| Fok et al. 2019 | Y | Y | Y | Y | Y | Y | Y | Y | Y | Y |
| Fuchs et al. (2020) | Y | Y | Y | Y | Y | Y | Y | Y | Y | Y |
| Galvis et al. (2014) | Y | Y | Y | Y | Y | Y | Y | Y | Y | Y |
| Gvozdenovic et al. (2020) | Y | N | Y | Y | Y | Y | Y | Y | Y | Y |
| Herzberg et al. 2010 | Y | Y | N | Y | Y | Y | N | N | Y | Y |
| Jawahier et al. (2023) | Y | Y | Y | Y | Y | Y | Y | Y | Y | Y |
| Kachooei et al. (2014) | Y | Y | Y | Y | Y | Y | Y | N | Y | Y |
| Kakar et al. (2012) | Y | Y | Y | Y | Y | Y | Y | Y | Y | Y |
| Lambrecht et al. (2022) | Y | Y | Y | Y | Y | Y | Y | Y | Y | Y |
| Laurentin-Perez et al. (2008) | Y | Y | Y | Y | Y | Y | Y | Y | Y | Y |
| Levina et al. (2023) | Y | Y | Y | Y | Y | Y | Y | Y | Y | Y |
| Maling et al. 2024 | Y | Y | Y | Y | Y | Y | Y | Y | Y | Y |
| Martínez Villén et al. (2023) | Y | Y | Y | Y | Y | Y | Y | Y | Y | Y |
| Mehling et al. (2023) | Y | Y | Y | Y | Y | Y | Y | Y | Y | Y |
| Rampazzo et al. (2015) | Y | Y | Y | Y | Y | Y | Y | Y | Y | Y |
| Reissner et al. (2016) | Y | Y | Y | Y | Y | Y | Y | Y | Y | Y |
| Sabo et al. (2014) | Y | Y | Y | Y | Y | Y | Y | Y | Y | Y |
| Saurbier 2013 | Y | Y | Y | Y | Y | Y | Y | N | Y | Y |
| Savvidou et al. (2013) | Y | Y | Y | Y | Y | Y | Y | Y | Y | Y |
| Scheker and Martineau 2013 | Y | Y | Y | Y | Y | Y | Y | Y | Y | Y |
| Scheker et al. 2008 | Y | Y | Y | Y | Y | N | Y | Y | Y | Y |
| Schuurman 2013 | Y | Y | Y | Y | Y | Y | Y | N | Y | Y |
| Shipley et al. (2009) | Y | Y | Y | Y | Y | Y | Y | N | Y | Y |
| Smith et al. (2024) | Y | Y | Y | Y | Y | Y | Y | Y | Y | Y |
| Stougie et al. (2023) | Y | Y | Y | Y | Y | Y | Y | Y | Y | Y |
| Stougie et al. (2023b) | Y | Y | Y | Y | Y | Y | Y | Y | Y | Y |
| Van Schoonhoven et al. (2012)  Van Schoonhoven et al. (2000) | Y | Y | Y | Y | Y | Y | Y | Y | Y | Y |
| Warlop et al. (2021) | Y | Y | Y | Y | Y | Y | Y | Y | Y | Y |
| Warwick et al. 2013 | Y | Y | Y | Y | Y | N | Y | Y | Y | Y |
| Willis et al. (2007) | Y | Y | Y | Y | Y | Y | Y | Y | Y | Y |
| Hebel et al. 2024 | Y |  |  |  |  |  |  |  |  |  |

**Online Table S3.** **Function results of the included studies.**

|  | | | DASH | | PRWE | | MWS | |
| --- | --- | --- | --- | --- | --- | --- | --- | --- |
|  |  |  | Mean postoperative score | Mean postoperative improvement from baseline | Mean postoperative score | Mean postoperative improvement from baseline | Mean postoperative score | Mean postoperative improvement from baseline |
| DRUJ Arthroplasty (APTIS) | | Number of studies (prostheses) | 9 studies (n=207) | 2 studies (n=23) | 11 studies (n=358) | 1 study (n=46) | 6 studies (n=113) | 4 studies (n=84) |
|  |  | Range (weighted mean) | 16-40 (25.4) | 8-51 (35.3) | 22-47 (30.4) | 34 (26) | 65-83 (69.9) | 15-53 (23.8) |
|  |  | Statistically significant? | - | In 1 out of 2 studies | - | Yes | - | In 3 out of 4* studies |
|  |  | Clinically significant? | - | In both studies | - | Yes | - | In all 4 studies |
| Ulnar Head Arthroplasty | All prostheses | Number of studies (prostheses) | 8 studies (n=234) | 4 studies (n=124) | 4 studies (n=151) | - | 6 studies (n=272) | 3 studies (n=167) |
|  |  | Range (weighted mean) | 5.9-43 (28.1) | 11-37 (19.6) | 12-52 (40.4) | - | 51-71 (62.0) | 14-36 (20.6) |
|  |  | Statistically significant? | - | In 3 out of 4* studies | - | - | - | In all 3 studies |
|  |  | Clinically significant? | - | In all 4 studies | - | - | - | In all 3 studies |
|  | Herbert UHP | Number of studies (prostheses) | 3 studies (n=100) | 2 studies (n=79) |  | - | 3 studies (n=185) | - |
|  |  | Range (weighted mean) | 27-43 (39.3) | 13-36 (37.9) |  | - | 61.8-71 (65.2) | - |
|  |  | Statistically significant? | - | In both studies |  | - | - | - |
|  |  | Clinically significant? | - | In both studies |  | - | - | - |
|  | uHead | Number of studies (prostheses) | - | - | - | - | - | 2 studies (n=65) |
|  |  | Range (weighted mean) | - | - | - | - | - | 29-36 (31.0) |
|  |  | Statistically significant? | - | - | - | - | - | In both studies |
|  |  | Clinically significant? | - | - | - | - | - | In both studies |

In statistical significance cells, number of denominator is different to total studies of that section because not all of them performed statistical significance tests.

**Online Table S4.** **Pain, satisfaction and grip strength results of the included studies.**

|  | | | Pain | | Satisfaction | Grip Strength | | |
| --- | --- | --- | --- | --- | --- | --- | --- | --- |
|  |  |  | Mean postoperative score (points) | Mean postoperative improvement from baseline (points) | Mean postoperative score | Mean postoperative score (kg) | Mean postoperative score (% contralateral hand) | Mean postoperative improvement from baseline (kg or % contralateral hand) |
| DRUJ Arthroplasty (APTIS) | | Number of studies (prostheses) | 17 studies (n=505) | 14 studies (n=421) | 5 studies (n=124) | 15 studies (n=446) | 9 studies (n=232) | 9 studies (n=192) |
|  |  | Range (weighted mean) | 1.0-3.9 (2.1) | 1.2-8.0 (4.3) | 79.7-100% (87.1%) | 8.2-35.8 (20.7) | 63.4-94% (75.7 | 0.3-23.7kg (8.0kg) |
|  |  | Statistically significant? | - | In 9 out of 11 studies | - | - | - | 4 out of 5 studies |
|  |  | Clinically significant? | - | In 10 out of 11 studies | - | - | - | - |
| Ulnar Head Arthroplasty | All prostheses | Number of studies (prostheses) | 7 studies (n=291) | 9 studies (n=323) | - | 8 studies (n=334) | 9 studies (n=275) | 6 studies (n=225) for kg  3 studies (n=59) for % contralateral hand |
|  |  | Range (weighted mean) | 0-2.5 (1.9) | 1.7-5.0 (2.5) | - | 14-35 (21.0) | 53.8-99% (79.9%) | 1-9kg (3.5kg)  -2-39.7% (20.6%) |
|  |  | Statistically significant? | - | In 6 out of 6 studies | - | - | - | 3 out of 6 studies |
|  |  | Clinically significant? | - | In all 9 studies | - | - | - | - |
|  | Herbert UHP | Number of studies (prostheses) | 3 studies (n=106) | 2 studies (n=85) | - | - | 4 studies (n=114) | 2 studies (n=40) |
|  |  | Range (weighted mean) | 1.7-2.5 (2.2) | 2.0-4.2 (3.6) | - | - | 53.8-90.8 (85.4%) | 20-39.7% (31.3%) |
|  |  | Statistically significant? | - | In both studies | - | - | - | In both studies |
|  |  | Clinically significant? | - | In both studies | - | - | - | - |
|  | uHead | Number of studies (prostheses) | 2 studies (n=66) | 2 studies (n=66) | - | 2 studies (n=66) | - | - |
|  |  | Range (weighted mean) | 1.6-2.0 (1.7) | 3.0-5.0 (3.6) | - | 21-21 (21) | - | - |
|  |  | Statistically significant? | - | In both studies | - | - | - | - |
|  |  | Clinically significant? | - | In both studies | - | - | - | - |

In statistical significance cells, number of denominator is different to total studies of that section because not all of them performed statistical significance tests. Clinical significance assessment was only conducted for pain which was a primary outcome.

*11^th^ study not statistically significant likely due to type 2 error (small population).

**Online Table S5.** **Supination and pronation range of movement results of the included studies.**

|  | | | Supination | | Pronation | |
| --- | --- | --- | --- | --- | --- | --- |
|  |  |  | Mean postoperative score (degrees) | Mean postoperative improvement from baseline (degrees) | Mean postoperative score (degrees) | Mean postoperative improvement from baseline (degrees) |
| DRUJ Arthroplasty (APTIS) | | Number of studies (prostheses) | 18 studies (n=512) | 12 studies (n=340) | 18 studies (n=512) | 12 studies (n=340) |
|  |  | Range (weighted mean) | 64-88 (76.1) | 4-25 (13.3) | 51-90 (69.6) | 2-20.3 (15.8) |
|  |  | Statistically significant? | - | In 5 out of 9 studies | - | In 5 out of 9 studies |
|  |  | Clinically significant? | - |  | - |  |
| Ulnar Head Arthroplasty | All prostheses | Number of studies (prostheses) | 12 studies (n=460) | 11 studies (n=365) | 12 studies (n=460) | 11 studies (n=365) |
|  |  | Range (weighted mean) | 65-83.1 (71.3) | -6-14.9 (2.6) | 60-76 (61.1) | -6-30.7 (3.7) |
|  |  | Statistically significant? | - | In 2 out of 9 studies | - | In 1 out of 9 studies |
|  |  | Clinically significant? | - | - | - | - |
|  | Herbert UHP | Number of studies (prostheses) | 6 studies (n=235) | 5 studies (n=225) | 6 studies (n=235) |  |
|  |  | Range (weighted mean) | 65-83.1 (71.3) | 0-14.9 (3.3) | 69-80.9 (64.1) | 1-30.7 (7.1) |
|  |  | Statistically significant? | - | In 1 out of 3 studies | - | In 1 out of 3 studies |
|  |  | Clinically significant? | - | - | - | - |
|  | uHead | Number of studies (prostheses) | 3 studies (n=85) | 3 studies (n=85) | 3 studies (n=85) | 3 studies (n=85) |
|  |  | Range (weighted mean) | 71-74 (72.1) | -6-0 (-3.9) | 60-73 (69.0) | -6- -0.8 (-4.4) |
|  |  | Statistically significant? | - | In none | - | In none |
|  |  | Clinically significant? | - | - | - | - |

In statistical significance cells, number of denominator is different to total studies of that section because not all of them performed statistical significance tests.

**Online Table S6.** **Pooled survival results of the included studies.**

|  | | | Survival | | | Overall complications | Re-operations | Deep infection | Superficial infection |
| --- | --- | --- | --- | --- | --- | --- | --- | --- | --- |
|  |  |  | Short-term (<4 years) | Mid-term (4-7 years) | Long-term (>7 years) |  |  |  |  |
| APTIS | | Number of studies (population) | 12 studies (n=417) | 7 studies (n=233) | 2 studies (n=31) | 18 studies (n=600) | 18 studies (n=600) | N=7 cases across studies (1.1%) | N=24 cases across studies (3.7%) |
|  |  | Range (weighted mean) | 90-100% (96.3%) | 80-100% (98.0%) | 84-100% (89.2%) | 12-83.3% (30.2%) | 6.1-47% (24.9%) |  |  |
| Ulnar head arthroplasty | Overall | Number of studies (population) | 6 studies (n=88) | 9 studies (n=523) | 4 studies (n=208) | 14 studies (n=640) | 17 studies (n=700) | N=7 cases across studies (1.1%) | N=2 cases across studies (0.3%) |
|  |  | Range (weighted mean) | 70-100% (89.8%) | 83-100% (94.6%) | 83-100% (87.3%) | 8/9-35.3% (22.4%) | 0-41.9% (22.6%) |  |  |
|  | Herbert UHP | Number of studies (population) | - | 2 studies (n=249) | 4 studies (n=208) | 6 studies (n=365) | 7 studies (n=467) |  | |
|  |  | Range (weighted mean) | - | 88.3-99% (98.3%) | 83-100% (87.3%) | 13-29% (20.0%) | 11-34% (18.2%) |  |  |
|  | Avanta uHead | Number of studies (population) | - | 2 studies (n=66) | - | 3 studies (n=85) | 3 studies (n=85) |  |  |
|  |  | Range (weighted mean) | - | 83-85% (83.6%) | - | 15-35.3% (27.7%) | 15-30% (23.7%) |  |  |

**Online Table S7. Main characteristics and details of the included prostheses**

|  | Inventors (country) | Type | First described in literature | Components | Materials | Modularity | Cemented/uncemented | DRUJ Stability |
| --- | --- | --- | --- | --- | --- | --- | --- | --- |
| APTIS | Scheker (USA) | Bipolar DRUJ replacement (both ulnar head and radial sigmoid notch) | 2008 | Ulnar stems: 4 diameters and 4 lengths (=16 options in total)  Radial plate: 3 sizes  UHMW polyethylene ball  Radial plate cover  One locking cross pin or two radial plate cover screws | All component made of cobalt-chromium (roughened titanium coating to stem) | Modular | Uncemented | Semi-constrained design (radial plate cover and securing screw(s) replace TFCC function |
| Herbert UHP | Herbert (Germany) | Ulnar head replacement only | 2000 | Stems: 3 diameters, 3 collar sizes (20mm extended collar available for revisions/Darrach salvage)  Heads: 3 sizes | Titanium stem  Ceramic head (metallic also available for salvage of failed Sauve-Kapandji) | Modular | Uncemented | Ulnar-based capsuloretinacular soft tissue flap |
| Avanta uHead | Berger (USA) | Ulnar head replacement only | 2002 | Stems: 4 diameters, 4 collar sizes (20mm extended collar available for revisions/Darrach salvage)  Heads: 3 sizes | Cobalt-chromium stem and head (roughened titanium coating to stem) | Modular | Uncemented | Slot for TFCC repair |
| Ascencion First Choice | Tagil, Kopylov, Beckenbaugh, Stanley (Sweden, USA, UK) | Ulnar head replacement only | 2011 | Stems: 3 diameters, 3 collar sizes  Heads: 3 sizes | Cobalt-chromium stem and head (roughened titanium coating to stem) | Total head modular  Partial head monoblock | Uncemented | Ulnar-based capsuloretinacular soft tissue flap for total  Preservation of TFCC/ulnar styloid for partial |
| Ascension MCP pyrocarbon (off-licence) | Used off-licence by the Mayo group, USA | Ulnar head replacement only | 2024 | Proximal component of MCP replacement (5 sizes) | Pyrocarbon | Monoblock | Uncemented | Preservation of TFCC/ulnar styloid |

*MCP, metacarpophalangeal; TFCC triangular fibrocartilage complex.*

**Identification of studies via databases and registers**

Records removed *before screening*:

Duplicate records removed (n = 435)

Records removed for other reasons (n = 0)

Records identified from:

Databases (n = 1046)

**Identification**

Records screened (title and abstract)

(n = 611)

Records excluded

(n = 5)

Reports sought for retrieval

(n = 68)

Reports not retrieved

(n = 0)

**Screening**

Reports excluded:

Reviews (n = 4)

Case reports or case series with less than 10 cases (n = 12)

Specific populations (n = 2)

Silicone arthroplasty (n = 2)

Reports assessed for eligibility

(n = 65)

Studies included in review

(n = 43)

(n = 45 articles)

**Included**

**Online Figure S1. PRISMA flow diagram**

Source: Page MJ, et al. BMJ 2021;372:n71. doi: 10.1136/bmj.n71.

This work is licensed under CC BY 4.0. To view a copy of this license, visit <https://creativecommons.org/licenses/by/4.0/>

| **Section and Topic** | **Item #** | **Checklist item** | **Location where item is reported** |
| --- | --- | --- | --- |
| **TITLE** | | |  |
| Title | 1 | Identify the report as a systematic review. | P1 |
| **ABSTRACT** | | |  |
| Abstract | 2 | See the PRISMA 2020 for Abstracts checklist. | P1 |
| **INTRODUCTION** | | |  |
| Rationale | 3 | Describe the rationale for the review in the context of existing knowledge. | P2, Lines 40-55 |
| Objectives | 4 | Provide an explicit statement of the objective(s) or question(s) the review addresses. | P2, Lines 56-58 |
| **METHODS** | | |  |
| Eligibility criteria | 5 | Specify the inclusion and exclusion criteria for the review and how studies were grouped for the syntheses. | P3, lines 71-75, P4 lines 95-105 |
| Information sources | 6 | Specify all databases, registers, websites, organisations, reference lists and other sources searched or consulted to identify studies. Specify the date when each source was last searched or consulted. | P3, Lines 63-69 |
| Search strategy | 7 | Present the full search strategies for all databases, registers and websites, including any filters and limits used. | P3, Lines 63-69 |
| Selection process | 8 | Specify the methods used to decide whether a study met the inclusion criteria of the review, including how many reviewers screened each record and each report retrieved, whether they worked independently, and if applicable, details of automation tools used in the process. | P3, Lines 75-76 |
| Data collection process | 9 | Specify the methods used to collect data from reports, including how many reviewers collected data from each report, whether they worked independently, any processes for obtaining or confirming data from study investigators, and if applicable, details of automation tools used in the process. | P3-4, Lines 90-93 |
| Data items | 10a | List and define all outcomes for which data were sought. Specify whether all results that were compatible with each outcome domain in each study were sought (e.g. for all measures, time points, analyses), and if not, the methods used to decide which results to collect. | P3, Lines 79-89 |
|  | 10b | List and define all other variables for which data were sought (e.g. participant and intervention characteristics, funding sources). Describe any assumptions made about any missing or unclear information. | P4, Lines 92-94 |
| Study risk of bias assessment | 11 | Specify the methods used to assess risk of bias in the included studies, including details of the tool(s) used, how many reviewers assessed each study and whether they worked independently, and if applicable, details of automation tools used in the process. | P4, Lines 107-109 |
| Effect measures | 12 | Specify for each outcome the effect measure(s) (e.g. risk ratio, mean difference) used in the synthesis or presentation of results. | P4, Lines 110-114 |
| Synthesis methods | 13a | Describe the processes used to decide which studies were eligible for each synthesis (e.g. tabulating the study intervention characteristics and comparing against the planned groups for each synthesis (item #5)). | P4 lines 95-105 |
|  | 13b | Describe any methods required to prepare the data for presentation or synthesis, such as handling of missing summary statistics, or data conversions. | P4, Lines 110-116 |
|  | 13c | Describe any methods used to tabulate or visually display results of individual studies and syntheses. | Tables 3-6 |
|  | 13d | Describe any methods used to synthesize results and provide a rationale for the choice(s). If meta-analysis was performed, describe the model(s), method(s) to identify the presence and extent of statistical heterogeneity, and software package(s) used. | N/A |
|  | 13e | Describe any methods used to explore possible causes of heterogeneity among study results (e.g. subgroup analysis, meta-regression). | N/A |
|  | 13f | Describe any sensitivity analyses conducted to assess robustness of the synthesized results. | N/A |
| Reporting bias assessment | 14 | Describe any methods used to assess risk of bias due to missing results in a synthesis (arising from reporting biases). | N/A |
| Certainty assessment | 15 | Describe any methods used to assess certainty (or confidence) in the body of evidence for an outcome. | N/A |
| **RESULTS** | | |  |
| Study selection | 16a | Describe the results of the search and selection process, from the number of records identified in the search to the number of studies included in the review, ideally using a flow diagram. | PRISMA flow chart |
|  | 16b | Cite studies that might appear to meet the inclusion criteria, but which were excluded, and explain why they were excluded. | P5, Lines 127-131 |
| Study characteristics | 17 | Cite each included study and present its characteristics. | Table 1, reference list |
| Risk of bias in studies | 18 | Present assessments of risk of bias for each included study. | Table 2 |
| Results of individual studies | 19 | For all outcomes, present, for each study: (a) summary statistics for each group (where appropriate) and (b) an effect estimate and its precision (e.g. confidence/credible interval), ideally using structured tables or plots. | Tables 3-6, PP 6-10 |
| Results of syntheses | 20a | For each synthesis, briefly summarise the characteristics and risk of bias among contributing studies. | Table 2 |
|  | 20b | Present results of all statistical syntheses conducted. If meta-analysis was done, present for each the summary estimate and its precision (e.g. confidence/credible interval) and measures of statistical heterogeneity. If comparing groups, describe the direction of the effect. | N/A |
|  | 20c | Present results of all investigations of possible causes of heterogeneity among study results. | N/A |
|  | 20d | Present results of all sensitivity analyses conducted to assess the robustness of the synthesized results. | N/A |
| Reporting biases | 21 | Present assessments of risk of bias due to missing results (arising from reporting biases) for each synthesis assessed. | N/A |
| Certainty of evidence | 22 | Present assessments of certainty (or confidence) in the body of evidence for each outcome assessed. | N/A |
| **DISCUSSION** | | |  |
| Discussion | 23a | Provide a general interpretation of the results in the context of other evidence. | P11, Lines 287-298 |
|  | 23b | Discuss any limitations of the evidence included in the review. | P12, Lines 332-343 |
|  | 23c | Discuss any limitations of the review processes used. | P12, Lines 332-343 |
|  | 23d | Discuss implications of the results for practice, policy, and future research. | P12, Lines 344-347 |
| **OTHER INFORMATION** | | |  |
| Registration and protocol | 24a | Provide registration information for the review, including register name and registration number, or state that the review was not registered. | Line 62 |
|  | 24b | Indicate where the review protocol can be accessed, or state that a protocol was not prepared. | Line 62 |
|  | 24c | Describe and explain any amendments to information provided at registration or in the protocol. | N/A |
| Support | 25 | Describe sources of financial or non-financial support for the review, and the role of the funders or sponsors in the review. | Title page |
| Competing interests | 26 | Declare any competing interests of review authors. | Title page |
| Availability of data, code and other materials | 27 | Report which of the following are publicly available and where they can be found: template data collection forms; data extracted from included studies; data used for all analyses; analytic code; any other materials used in the review. | Title page |

**Online Figure S2. PRISMA checklist**
